# Cervical spondylotic internal jugular venous compression syndrome

**DOI:** 10.1111/cns.13148

**Published:** 2019-05-22

**Authors:** Jia‐Yue Ding, Da Zhou, Li‐Qun Pan, Jing‐Yuan Ya, Cheng Liu, Feng Yan, Chun‐Qiu Fan, Yu‐Chuan Ding, Xun‐Ming Ji, Ran Meng

**Affiliations:** ^1^ Department of Neurology Xuanwu Hospital, Capital Medical University Beijing China; ^2^ Advanced Center of Stroke Beijing Institute for Brain Disorders Beijing China; ^3^ Department of China‐America Institute of Neuroscience Xuanwu Hospital, Capital Medical University Beijing China; ^4^ Department of Neurology Yongxin People’s Hospital Ji’an China; ^5^ Department of Neurosurgery Xuanwu Hospital, Capital Medical University Beijing China; ^6^ Department of Neurosurgery Wayne State University School of Medicine Detroit Michigan

**Keywords:** cerebral venous stenosis, cervical spondylosis, internal jugular vein, osseous compression

## Abstract

**Aims:**

This study aimed to identify the clinical profiles of cervical spondylosis‐related internal jugular vein stenosis (IJVS) comprehensively.

**Methods:**

A total of 46 patients, who were diagnosed as IJVS induced by cervical spondylotic compression were recruited. The clinical manifestations and imaging features of IJVS were presented particularly in this study.

**Results:**

Vascular stenosis was present in 69 out of the 92 internal jugular veins, in which, 50.7% (35/69) of the stenotic vessels were compressed by the transverse process of C1, and 44.9% (31/69) by the transverse process of C1 combined with the styloid process. The transverse process of C1 compression was more common in unilateral IJVS (69.6% vs 41.3%, *P* = 0.027) while the transverse process of C1 combined with the styloid process compression had a higher propensity to occur in bilateral IJVS (52.2% vs 30.4%, *P* = 0.087). A representative case underwent the resection of the elongated left lateral mass of C1 and styloid process. His symptoms were ameliorated obviously at 6‐month follow‐up.

**Conclusions:**

This study proposes cervical spondylotic internal jugular venous compression syndrome as a brand‐new cervical spondylotic subtype. A better understanding of this disease entity can be of great relevance to clinicians in making a proper diagnosis.

## 1 INTRODUCTION

Cervical spondylosis is a commonly encountered degenerative disorder of the cervical spine. Abnormal structures, repetitive movements, and aging degeneration can exacerbate bone hyperplasia and losses of intervertebral disk contents and subsequently cause the disk to bulge outward, which in turn hasten the damage to the disk and surrounding structures gradually.^1^ The pathophysiological changes following cervical spondylosis may contribute to a plethora of clinical manifestations, such as cervical radiculopathy, cervical myelopathy, axial neck or shoulder pain, and vertebral artery insufficiency. Previous studies mainly focused on the implication of the spinal cord, nerve roots, and vertebral arteries, whereas, in our clinical practice, we have noticed that a subset of patients with unexplained nonfocal neurological dysfunctions display cerebral venous outflow disturbance in relation to the atlas (C1) compression of the superior segment of the internal jugular vein (IJV).^1^

Although current understanding of IJV stenosis (IJVS) is far from adequate, IJVS has been demonstrated to be associated with several central nervous system (CNS) disorders, including transient monocular blindness, Ménière disease, Alzheimer's disease (AD), idiopathic intracranial hypertension, and multiple sclerosis.^2^, ^3^, ^4^, ^5^, ^6^, ^7^, ^8^ Accumulating evidence also reveals that extracranial venous outflow disturbance poses an undesirable effect on the cerebral arterial and venous circulation, thus resulting in varied neurological deficits.^9^ Decreased cerebral perfusion, cerebral microvascular structures impairment, impaired cerebrospinal fluid (CSF) dynamics, and elevated intracranial pressure may be the underlining mechanisms of IJVS‐induced brain structural and functional disorders.^10^ Our study group discovered that some nonfocal neurological symptoms like headache, head noise, tinnitus, and visual impairment are tightly correlated to unilateral or bilateral IJVS, and balloon dilation with stenting in the stenotic segment may be a promising option to overcome nonimmunogenic and nonextrinsic compression IJVS‐induced jugular venous outflow impairment.^11^ Meanwhile, we also depicted the clinical characteristics and neuroimaging findings in IJVS in a previous publication.^12^ Accordingly, symptomatic IJVS should be viewed as a pathological disorder, which deserves more attention from clinicians due to the limited realization and the disabling or long‐lasting neurological symptoms that cannot be explained by other known diseases.^13^

The IJV can be thought of as having three segments: the inferior (J1), mid (J2), and superior segments (J3). Given the anatomical fact that the J3 segment passes through the interval space between the C1 lateral tubercle and the styloid process, this part is more likely to be impinged by osseous structures from cervical spondylosis.^9^, ^14^, ^15^ In this study, we aimed to identify the clinical profiles of cervical spondylosis‐related IJVS in attempt to gain a deeper understanding of the negative impact of cervical spondylotic IJV compression syndrome on CNS.

## 2 METHODS

This study was one part of a prospective study registered on Clinical Trial Registration (NCT03373292) and was approved by the Institutional Ethic Committee of Xuanwu Hospital, Capital Medical University (Beijing, China) in accordance with the guidelines of the 1964 Declaration of Helsinki. Informed consent was obtained from all individuals before collecting any data. From November 2017 through July 2018, a total of 46 patients, who were diagnosed with cervical spondylotic IJV compression syndrome in the departments of Neurology and Neurosurgery of Xuanwu Hospital, Capital Medical University, were enrolled in this study. The diagnosis of cervical spondylotic IJVS was confirmed by magnetic resonance venography (MRV) and/or computed tomographic venography (CTV). Other imaging modalities, including catheter venography (CV), magnetic resonance imaging (MRI), cervical duplex ultrasound, and single‐photon emission computed tomography (SPECT), were conducted in a portion of the patients as well. All the imaging tests were performed in the supine position.

Patients with cervical spondylotic IJVS should comply with each item as follows: (a) the stenotic segment narrowing of ≥50% in respect to the proximal adjacent jugular vein segment, as shown in MRV, CTV, or CV; (b) at least one abnormal collateral vessel ≥50% of the maximal diameter of the adjacent IJV or at least two abnormal collateral vessels <50% of the maximal diameter of the adjacent IJV, as depicted by MRV, CTV, or CV; (c) IJVS secondary to the compression from the cervical lateral mass with or without the styloid process, as depicted by CTV; (d) with unexplained nonfocal neurological deficits or other symptoms.^10^, ^16^, ^17^

All patients received standard medical therapy, including antiplatelets/anticoagulation, dehydration, and other symptomatic treatment to prevent against venous thrombosis, decrease intracranial hypertension, and relieve afflicted symptoms.^10^, ^12^ As there is no consensus over the surgical intervention for such osseous impingement, only patients who strongly requested surgery were performed with cervical lateral mass resection and subsequent stenting/balloon dilatation.

SPSS 19.0 was used in this study for data analysis. Continuous variables following Gaussian distribution were expressed as mean ± standard deviation (SD) and analyzed by *t* test or one‐way analysis of variance (ANOVA); otherwise, they were presented as median (interquartile range, IQR) and analyzed by Mann‐Whitney *U* test. Categorical variables were depicted as number (percentage) and analyzed by chi‐square test. A *P*‐value < 0.05 was considered as statistical significance.

## 3 RESULTS

### 3.1 Population and demographics

Between November 2017 and July 2018 in the departments of Neurology and Neurosurgery of Xuanwu Hospital (Beijing, China), a total of 118 patients diagnosed with IJVS were consecutively enrolled into this prospective study. In accordance with the diagnostic criteria of cervical spondylotic IJVS, 72 cases including 51 cases of IJVS without extrinsic compression, 19 cases of IJVS with extrinsic compression from the sternocleidomastoid muscle, enlarged thyroid glands, lymph glands, or carotid arteries, and two cases of IJVS with isolated styloidogenic compression, were excluded from this study. Accordingly, 46 (92 sides of the IJVs) including 20 male and 26 female patients were enrolled in the final analysis. The average age of the 46 cases was 57.4 ± 9.9 years (ranging from 28 to 74) and the average age of symptom onset was 46.4 ± 14.6 (ranging from 18 to 71) years. The median onset‐to‐door time was 7.5 (IQR, 3‐17.75) years. Details of the demographics are displayed in Table 1.

**Table 1:** Baseline clinical characteristics of the involved patients

Clinical features	
Demographics	
No. of patients	46
Gender (male/female)	20/26
Age (y)	57.4 ± 9.9
Symptoms onset age (y)	46.4 ± 14.6
Onset‐to‐door time (y)	7.5 (3‐17.75)
Comorbid diseases	
Hypertension	11 (23.9)
Diabetes	6 (13.0)
Hyperlipidemia	14 (30.4)
Coronary heart disease	8 (17.4)
Hepatopathy	5 (10.9)
Nephropathy	2 (4.3)
Traditional cervical spondylosis^a^	3 (6.5)
Cerebral hemorrhage	1 (2.2)
Cerebral infarction	8 (17.4)
Cerebral venous sinus thrombosis	1 (2.2)
Life habit	
Alcohol abuse	9 (19.6)
Smoking	6 (13.0)
Clinical manifestations	
Insomnia	31 (67.4)
Hearing impairment	22 (47.8)
Visual impairment	16 (34.8)
Headache	18 (39.1)
Tinnitus	32 (69.6)
Head noise	33 (71.7)
Dry eyes	16 (34.8)
Uncomfortable neck	14 (30.4)
Vertigo	9 (19.6)
Dizziness	25 (54.3)
Anxiety or depression	9 (19.6)
Nausea or vomiting	4 (8.7)
Memory deterioration	5 (10.9)
Median (IQR) number of manifestations	5 (4‐6)

a Traditional cervical spondylosis includes cervical radiculopathy, cervical myelopathy, axial neck pain and vertebral artery insufficiency.

### 3.2 Clinical features

Prominent clinical symptoms of cervical spondylotic IJV compression syndrome in the present study were as follows: head noise (33/46, 71.7%), tinnitus (32/46, 69.6%), insomnia (31/46, 67.4%), dizziness (25/46, 54.3%), hearing impairment (22/46, 47.8%), headache (18/46, 39.1%), visual impairment (16/46, 34.8%), dry eyes (16/46, 34.8%), uncomfortable neck (14/46, 30.4%), vertigo (9/46, 19.6%), anxiety or depression (9/46, 19.6%), memory deterioration (5/46, 10.9%), and nausea or vomiting (4/46, 8.7%). Other atypical symptoms included head numbness, palpitation, fainting, and dyspnea, each of which occurred in only one patient. The median number of manifestations was 5 (IQR, 4‐6) for the involved patients. Details are shown in Table 1.

With regard to the comorbid diseases, hyperlipidemia was found in 30.4% (14/46) patients, followed by hypertension (11/46, 23.9%), coronary heart disease (8/46, 17.4%), and cerebral infarction (8/46, 17.4%). There were only three cases accompanied by non‐IJVS cervical spondylosis (two cases with cervical radiculopathy and one case with cervical myelopathy).

### 3.3 Imaging characteristics

**Figure 1:**
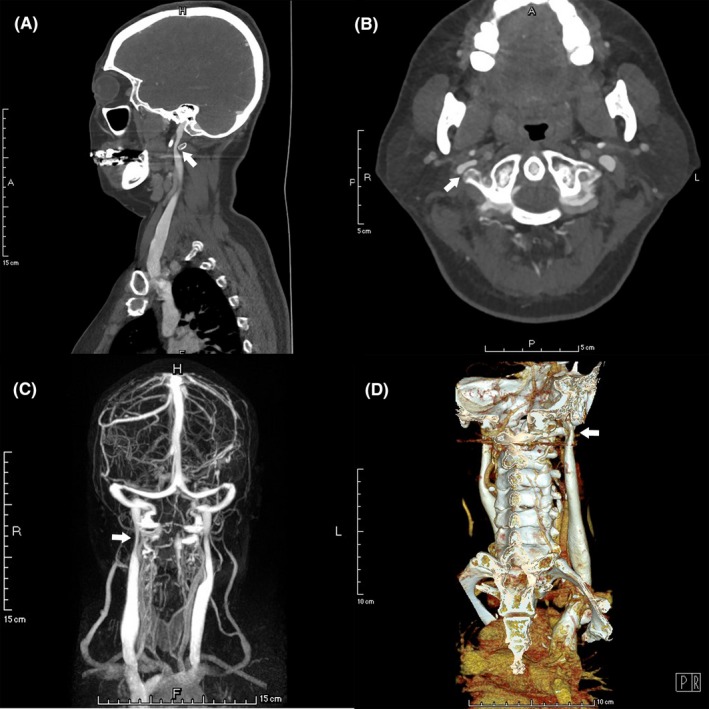
Neuroimaging features of right IJVS induced by the C1 transverse mass. Sagittal (A), axial (B), and 3D reconstructive (D) CTV images revealing the right transverse mass of C1 compression on the right IJV. MRV (C) revealing the right IJV‐J3 segment stenosis accompanied by substantially abnormal collateral veins

**Figure 2:**
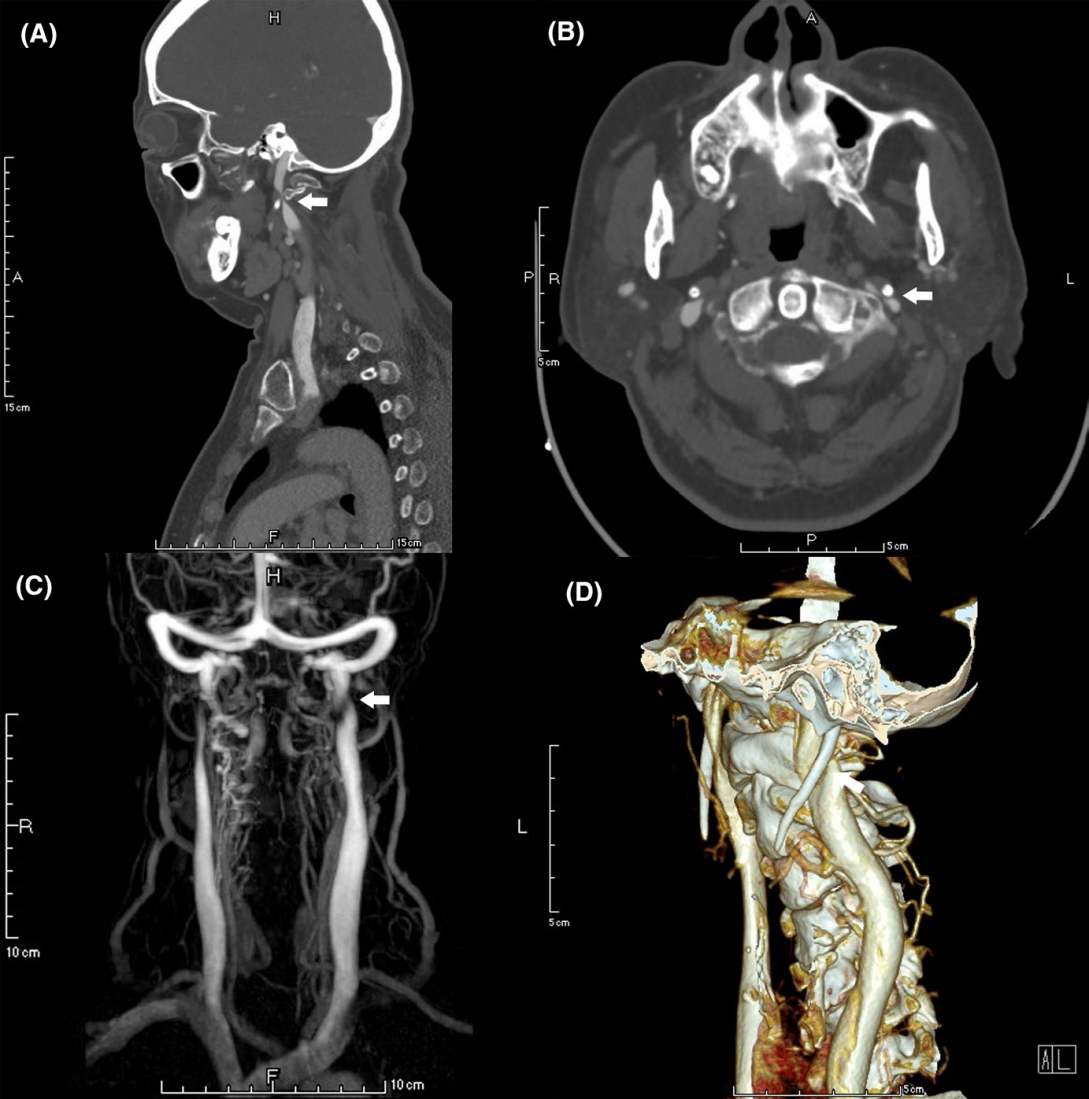
Neuroimaging features of left IJVS induced by the C1 transverse mass and styloid process. Sagittal (A), axial (B), and 3D reconstructive (D) CTV images revealing the left transverse mass of C1 combined with styloid process compression on the left IJV. MRV (C) revealing the left IJV‐J3 segment stenosis accompanied by substantially abnormal collateral veins

**Figure 3:**
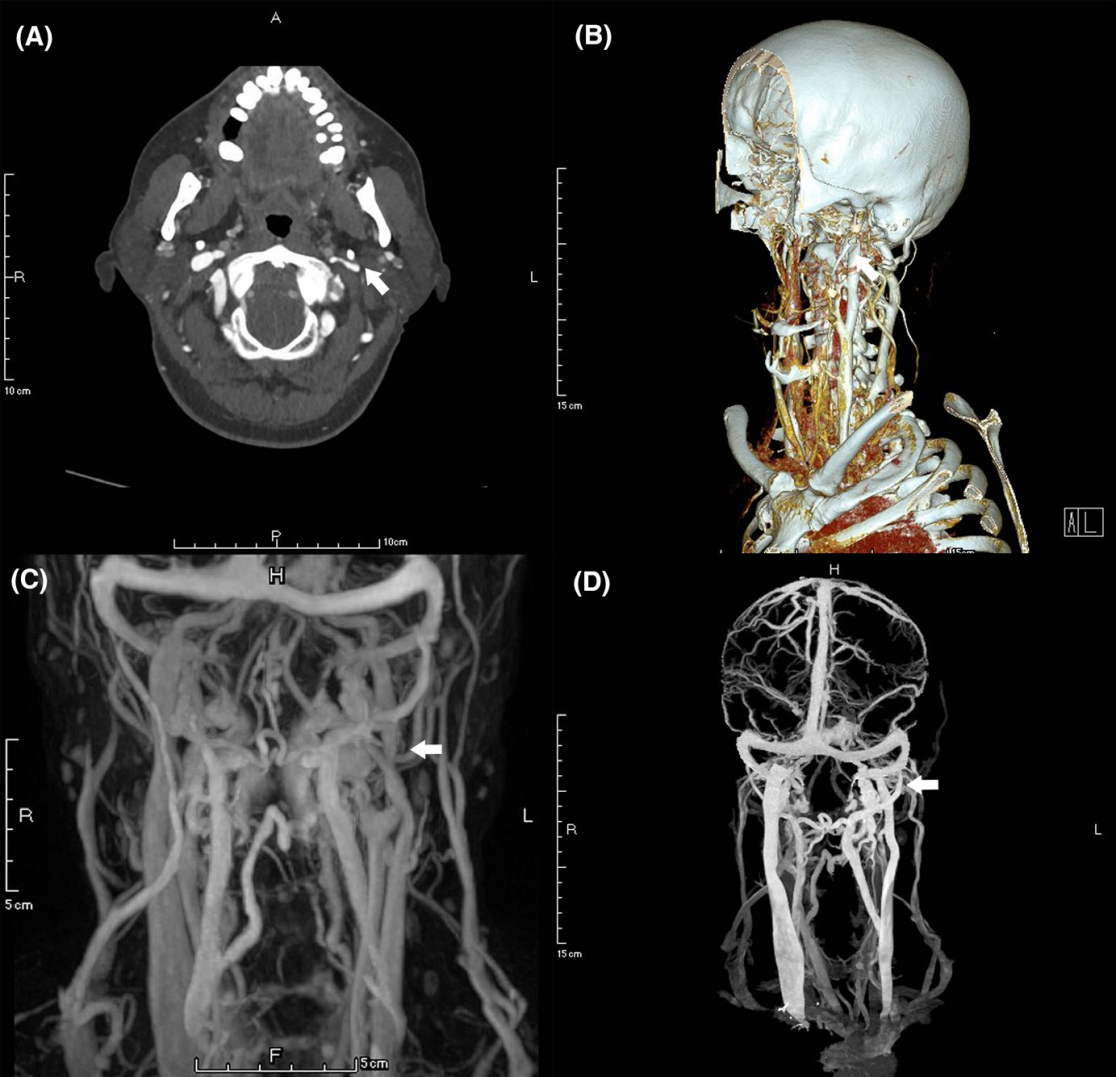
Neuroimaging features of left IJVS induced by the styloid process. Axial, (A) and 3D reconstructive (B) CTV images showing the left IJVS present the left styloid process compression on the left IJV. MRV (C) and CTV (D) showing the left IJV‐J3 segment stenosis accompanied by substantially abnormal collateral veins

Vascular stenosis was present in 69 out of the 92 IJVs, in which, 50.7% (35/69) was caused by the compression from the transverse process of C1 (Figure 1), 44.9% (31/69) by the compression from the transverse process of C1 combined with the styloid process (Figure 2), and 2.9% (2/69) by the styloid process (Figure 3). Only 1 (1.4%) stenotic vessel was not due to extrinsic compression. Meanwhile, 5 (7.2%) stenotic vessels were compressed by both the surrounding bony structures and carotid arteries. Of note, the majority of IJVS (68/69, 98.6%) occurred at J3 segment, followed by 5.8% (4/69) at J2 and 2.9% (2/69) at J1 segment. All the osseous impingement observed in the present study resulted from the lateral mass of C1 and/or the styloid process. The duplex ultrasound (supine position) revealed that the mean diameters of stenotic segments and nonstenotic segments were 3.94 ± 2.00 mm and 7.34 ± 2.26 mm, respectively (*P* < 0.001), and the median (IQR) volume flow for stenotic and nonstenotic segments were 125 (47.5‐190) and 220 (138.25‐292.5) mL/min (*P* = 0.029). The mean diameter of vertebral veins (VV) was 3.13 ± 0.84 mm (3.17 ± 0.83 mm for the stenotic side and 3.01 ± 0.86 mm for the nonstenotic side, *P* = 0.428). Representative IJVS imaging characteristics are presented in Table 2.

**Table 2:** Characteristics of IJVS on MRV, CTV and CV imaging

Imaging characteristics	
Stenotic segments (sides, %)	
J1	2 (2.9)
J2	4 (5.8)
J3	68 (98.6)
Stenotic sites (patients, %)	
Left	12 (26.1)
Right	11 (23.9)
Bilateral	23 (50.0)
Types of compression (sides, %)	
Transverse mass of C1 compression	35 (50.7)
Syloid process compression	2 (2.9)
Transverse mass of C1 combined with styloid process compression	31 (44.9)
Ultrasound (in the supine position)	
Average stenotic IJV segment diameter, mm	3.94 ± 2.00
Average nonstenotic IJV segment diameter, mm	7.34 ± 2.26
Average stenotic IJV segment volume flow, mL/min	125 (47.5‐190)
Average nonstenotic IJV segment volume flow, mL/min	220 (138.25‐292.5)
Average VV diameter, mm	3.13 ± 0.84

Note

Of the 69 stenotic vessels, 68 were secondary to extrinsic osseous compression (five stenotic vessels were also compressed by the nearby carotid arteries) and the remaining one stenotic vessel was not associated with external compression.

### 3.4 Clinical features between uni‐ and bilateral IJVS

As shown in Table 3, the percentage of patients with unilateral cervical spondylotic compression‐induced IJVS was nearly equivalent to that of bilateral compression‐induced IJVS. It was found that the C1 transverse process compression was more common in unilateral than that in bilateral IJVS (69.6% vs 41.3%, *P* = 0.027). In contrast, dual compression from the lateral mass of C1 combined with the styloid process was more prone to occur in bilateral IJVS compared with that in unilateral IJVS (52.2% vs 30.4%, *P* = 0.087). The clinical manifestations aforementioned in unilateral IJVS were well matched with that in bilateral IJVS subgroup. In addition, the average age of symptom onset and the average duration between symptom onset and admission to our clinical center were similar between two IJVS subgroups.

**Table 3:** Comparisons between unilateral and bilateral IJVS

	Unilateral stenosis	Bilateral stenosis	*P*‐value
Demographics			
No. of patients	23	23	NA
Age (y)	55.1 ± 9.4	59.6 ± 10.1	0.126
Symptom onset age (y)	43.8 ± 15.9	49.0 ± 12.9	0.229
Onset‐to‐door time (y)	7 (4‐20)	8 (3‐15)	0.741
Gender (male/female)	11/12	9/14	0.552
Types of compression on IJV (sides, %)			
Transverse mass of C1 compression	16 (69.6)	19 (41.3)	0.027
Transverse mass of C1 combined with styloid process compression	7 (30.4)	24 (52.2)	0.087
Styloid process compression	0 (0.0)	2 (4.3)	NA
Clinical manifestations			
Insomnia	15 (65.2)	16 (69.6)	0.753
Hearing impairment	9 (39.1)	13 (56.5)	0.238
Visual impairment	8 (34.8)	8 (34.8)	1.000
Headache	8 (34.8)	10 (43.5)	0.546
Tinnitus	15 (65.2)	17 (73.9)	0.522
Head noise	16 (69.6)	17 (73.9)	0.743
Dry eyes	5 (21.7)	11 (47.8)	0.063
Uncomfortable neck	10 (43.5)	4 (17.4)	0.055
Vertigo	4 (17.4)	5 (21.7)	1.000
Dizziness	11 (47.8)	14 (60.9)	0.375
Anxiety or depression	6 (26.1)	3 (13.0)	0.457
Nausea or vomiting	2 (8.7)	2 (8.7)	1.000
Memory deterioration	4 (17.4)	1 (4.3)	0.399
Median (IQR) number of manifestations	5 (3‐6)	5 (4‐7)	0.633

Abbreviation: NA, not applicable.

### 3.5 Outcomes of a representative case with bilateral IJVS who underwent bone resection and subsequent balloon dilation

Up to now, only 1 case in this cohort underwent bone resection and unilateral balloon dilation. This 49‐year‐old male patient who complained with headache, head noise, and dizziness for 3 years was diagnosed as cervical spondylotic compression‐induced bilateral IJVS in Xuanwu Hospital in November 2017. MRV showed significant stenosis at bilateral IJV‐J3 segments, surrounded by abnormally distorted collateral venous plexuses (Figure S1A). As demonstrated in CTV, bilateral IJV‐J3 segments were profoundly compressed by the adjacent lateral mass of C1 and styloid process (Figure S1B‐F). The jugular venous ultrasound at admission indicated that the internal diameter of the distal J3 segment was 1.9 mm and the flow velocity was 96 cm/s.

After excluding the presence of prominent comorbid diseases, this patient underwent surgical intervention. Left IJV was selected to treat with dilatation due to more severe outflow disturbance shown in CV. Both the left lateral mass of C1 and styloid process were resected and the left stenotic segment was corrected with balloon dilation. Two weeks later, although this patient still had head noise, his headache and dizziness were mildly ameliorated. Postsurgery CTV also revealed that the previous stenotic segment was corrected (Figure S2A‐F). At 4‐month follow‐up, the jugular venous ultrasound showed that the internal diameter of the distal J3 segment enlarged to 3.1 mm and the flow velocity decreased to 59 cm/s. Moreover, at 6‐month follow‐up, his headache and dizziness were markedly ameliorated, and his head noise completely disappeared.

## 4 DISCUSSION

Although traditional cervical spondylosis including radiculopathy, myelopathy, axial neck pain and vertebral artery insufficiency has been frequently reported in the literature, studies focusing on cervical spondylosis‐induced venous outflow disturbance are still lacking. In our clinical practice, we have noticed that a large percentage of IJVS patients display with osseous impingement or compression, with the first cervical vertebra (atlas, C1) being the major contributor. As the IJV passes over the anterior aspect of the transverse process of C1 and once the cervical vertebral structure changes, this IJV segment is likely to be compressed.^9^, ^10^, ^12^, ^13^, ^14^ Given that, we bring forward a novel concept: “cervical spondylotic internal jugular venous compression syndrome,” to depict the clinical presentations and imaging features in patients with this issue.

In the routine examination, narrowing of the jugular veins below the skull base is not an uncommon finding. Both clinical presentations and imaging features should be taken into consideration when clinicians attempt to make the diagnosis of IJVS^9^, ^10^, ^11^, ^12^. In this study, most of the patients with cervical spondylotic IJVS were at their middle age (57.4 ± 9.9 years), and the average age of symptom onset was approximately 10 years younger (46.4 ± 14.6 years). The ages of the cohort were consistent with the age predilection of traditional cervical spondylosis^1^. Long‐time working that involves repetitive neck motions or abnormal positioning may be the most vital risk factor for the deterioration of cervical vertebrae among young individuals, leading to osseous impingement‐induced IJVS.

To our knowledge, extracranial venous drainage impairment is associated with a number of brain disorders.^10^ How to better understand the pathophysiological mechanisms underlying IJVS due to varied etiologies has aroused a great interest. The clinical presentations in this cohort with osseous impingement are quite similar to a previously reported cohort of IJVS without external compression, including headache, head noise or tinnitus, sleeping disorder and even hearing or visual impairment, denoting that IJVS with different etiologies may share the same pathological mechanisms, irrespective of whether there exists external compression or not.^11^, ^12^ Importantly, stenosis and abnormal collateral veins shown by MRV or CTV are essential for the diagnosis of symptomatic IJVS.^17^

At this time, an inadequate understanding of symptoms and signs of cervical spondylotic IJVS may underestimate its clinical significance. All patients in this study had been misdiagnosed before they were admitted into our institution (the longest duration of misdiagnosis is 8 years). Therefore, in addition to reporting a special cervical spondylosis subtype‐cervical spondylotic IJVS, this study also provides a valuable reference for clinicians who are unfamiliar with this novel concept, whereby decreasing the rate of misdiagnosis and treatment delay.

The main reason for the focal stenosis at the IJV‐J3 segment was due to the transverse process with or without styloid process compression in this cohort of patients. Two patients with styloid process compression alone were ruled out from the analysis, as this issue does not belong to cervical spondylosis. Moreover, it has been reported that patients with styloidogenic IJVS might benefit from decompressive styloidectomy.^14^, ^15^ Other extrinsic or nonextrinsic stenoses were excluded from this study as well. Risk factors for nonextrinsic stenoses have not been determined currently, whereas, our clinical practice implies that certain systemic disorders, such as autoimmunity diseases, hypertension and diabetes may be associated with venous wall anomalies of the IJV.^10^, ^12^ Consequently, a total of 46 patients were involved. Of the 69 stenotic IJVs, 68 were identified as cervical spondylotic compression‐induced stenosis and the remaining one stenotic vessel was not associated with external compression. Meanwhile, two stenotic vessels resulted from the elongated styloid process alone and five stenotic vessels were due to both the surrounding bony structures and carotid arteries. Therefore, these findings indicate that the underlying etiologies for IJVS are varied (the cervical vertebra may not be the only one), and imaging examinations should be performed carefully. The duplex ultrasound further confirmed the diagnosis of IJVS, that is, the diameters and volume flow of the stenosed IJV segments presented in MRV/CTV/CV were significantly smaller than those of non‐stenotic segments. Meanwhile, the luminal diameters of bilateral VV were also normal, irrespective of the stenotic or nonstenotic side.^18^, ^19^, ^20^ These findings suggest that the vertebral system was fluent, and these symptoms and compensatory collateral development were mainly attributed to IJV anomalies.

Data from this study revealed that the percentage of patients with unilateral cervical spondylotic compression‐induced IJVS was nearly equivalent to that of bilateral compression‐induced IJVS. Notably, isolated compression from the transverse process was more likely in unilateral IJVS while dual compression from the transverse process combined with the styloid process was more common in bilateral IJVS. The progressively exacerbating feature of cervical spondylosis may contribute to the discrepancy. We hypothesize that mild cervical spondylosis may only result in unilateral IJV compression by the transverse process alone, whereas, bilateral IJV may be implicated eventually with the deterioration of this abnormality. Although bilateral IJVS are conceived as the late stage of cervical spondylotic IJVS, the clinical symptoms, the average age of symptom onset, and the average duration between symptom onset and admission to our institution did not differ from unilateral IJVS.

Previous studies have shown uncertain results regarding the effect of endovascular stenting on IJVS patients with multiple sclerosis.^21^, ^22^, ^23^ Our team reported that patients with nonextrinsic IJVS in the absence of multiple sclerosis or other intracranial pathologies could benefit from intravenous balloon angioplasty combined with stenting.^11^ However, with regard to the identified extrinsic impingement of the IJV, stenting alone was deemed ineffective and might even exacerbate the outflow disturbance.^14^ In this condition, decompressive bone resection may serve as a potential therapeutic strategy, which not only relieves IJVS‐associated symptoms but also lowers the complications of stenting.^14^ In this study, only one case underwent bone resection and unilateral balloon dilation. Despite the lack of long‐term follow‐up data, the functional outcome of this patient has been satisfactory so far. Accordingly, larger prospective trials exploring the efficacy and safety of surgical intervention in patients with cervical spondylotic IJVS are warranted.

There are several limitations in the study. First of all, the small sample size may bias the epidemiological results in this study. Second, the severity of some symptoms was not quantified. Given that, the exact difference between unilateral IJVS and bilateral IJVS could not be assessed accurately. Finally, only one case underwent surgical intervention without complete follow‐up data. Therefore, the efficacy and safety of bone resection could not be concluded in this study.

## 5 CONCLUSION

This study proposes cervical spondylotic IJV compression syndrome as a new cervical spondylosis subtype. A better understanding of the clinical presentations and imaging features of this type of cervical spondylosis can be of great relevance to clinicians in making a proper diagnosis. Compression from the transverse process of C1 alone was more common in unilateral IJVS, while dual compression from the transverse process combined with the styloid process was more likely in bilateral IJVS Moreover, the lateral tubercle and styloid process resection may be a promising option to address cervical spondylotic IJVS‐associated symptoms. Given the limitations mentioned above, well‐designed clinical trials with large sample size are required to further investigate this disorder.

## CONFLICT OF INTEREST

Jiayue Ding, Da Zhou, Liqun Pan, Jingyuan Ya, Cheng Liu, Feng Yan, Chunqiu Fan, Yuchuan Ding, Xunming Ji and Ran Meng declare that they have no conflicts of interest with the contents of this article.

## AUTHOR CONTRIBUTIONS

JD and DZ performed all the statistical analyses with the guidance of the department of statistics of Capital Medical University and drafted the manuscript. LP, JY, CF, and FY selected appropriate subjects from Xuanwu Hospital. RM, YD, DZ, and JD reviewed and edited the manuscript. CL and DJ provided figures in this study. XJ and RM contributed to the conception and design of this study and proposed the amendments.
