# Supplementary figures and images for: Cervical spondylotic internal jugular venous compression syndrome
Source: CNS Neurosci Ther. 2019 May 22;26(1):47–54. doi: 10.1111/cns.13148 (PMC6930831; doi:10.1111/cns.13148)

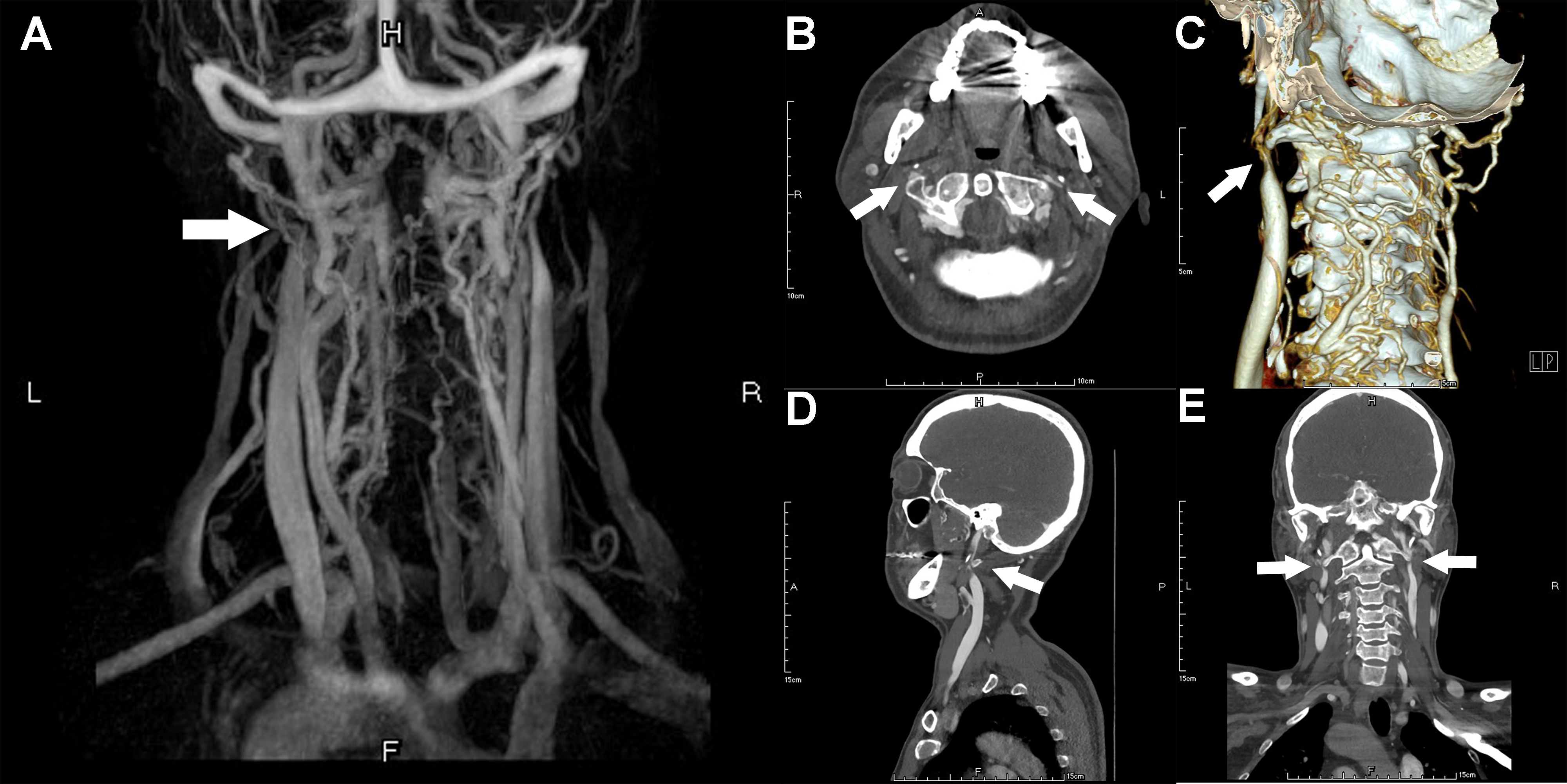

Supplement: Supplementary file 1 [file CNS-26-47-s001.tif]

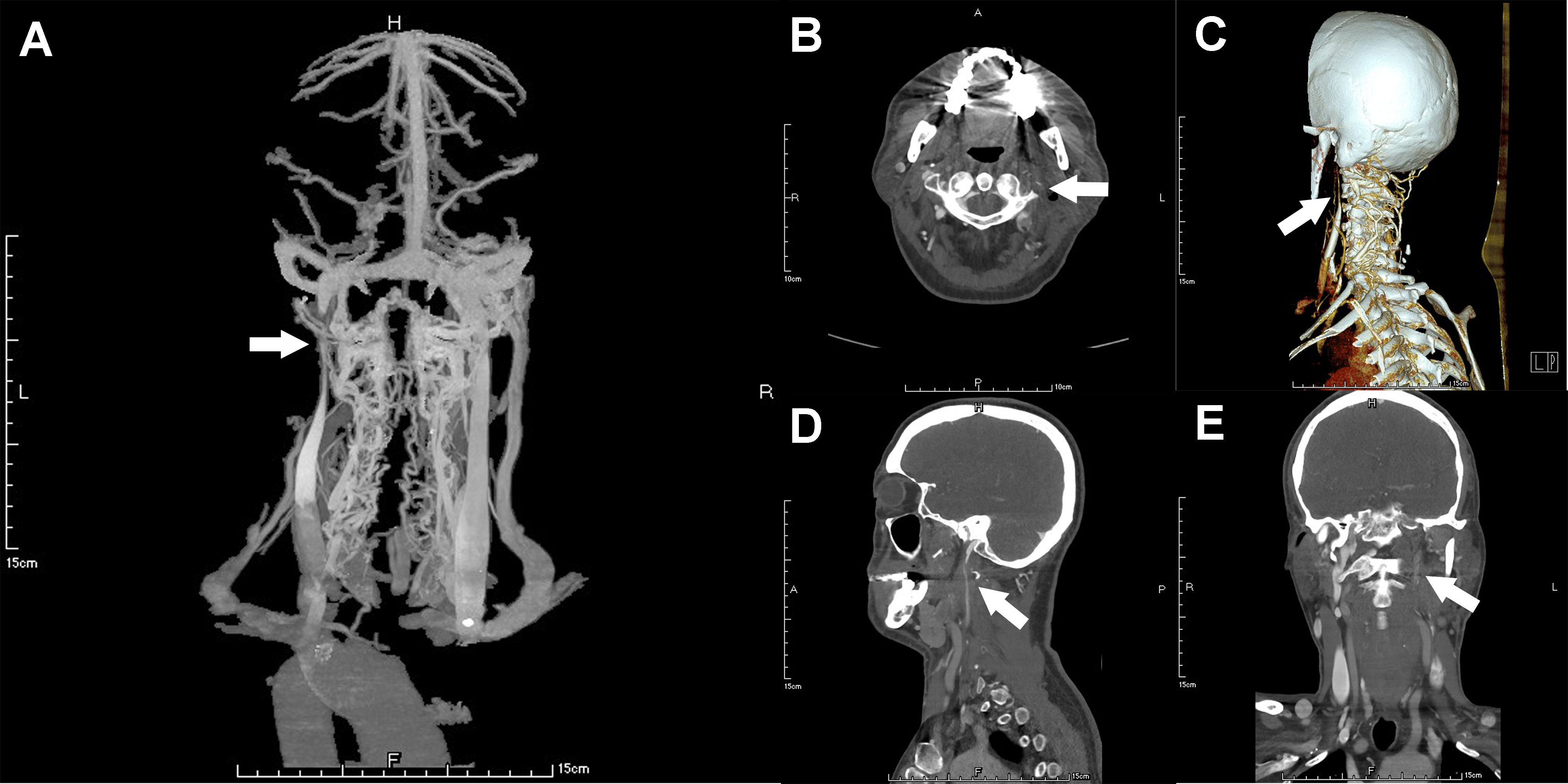

Supplement: Supplementary file 2 [file CNS-26-47-s002.tif]
