# Supplementary material for: Cervical spondylotic internal jugular venous compression syndrome
Source: CNS Neurosci Ther. 2019 May 22;26(1):47–54. doi: 10.1111/cns.13148 (PMC6930831; doi:10.1111/cns.13148)
Supplement: Supplementary file 3 [file CNS-26-47-s003.docx]

Figure S1. Preoperative neuroimaging features of bilateral IJVS. Preoperative MRV (A) shows bilateral IJVS at J3 segment, surrounded by substantially abnormal collateral veins. Axial, sagittal, coronal, and 3D reconstructive CTV images (B-E) show that the J3 segment of the left IJV is compressed by the left transverse mass of C1 and the styloid process.

Figure S2. Postoperative neuroimaging features of IJVS. Postoperative CTV (A) shows the mildly dilated left IJV-J3 segment surrounding by substantial abnormal collateral veins. Axial, sagittal, coronal, and 3D reconstructive CTV images (B-E) show the compression from the left transverse mass of C1 and the styloid process is absent, and the diameter of the left IJV-J3 segment is mildly increased.
